# Cytoreductive Surgery Plus Hyperthermic Intraperitoneal Chemotherapy for Patients with Peritoneal Metastases from Endometrial Cancer

**DOI:** 10.1245/s10434-017-6307-3

**Published:** 2017-12-27

**Authors:** Tommaso Cornali, Paolo Sammartino, Nikolaos Kopanakis, Athina Christopoulou, Marialuisa Framarino dei Malatesta, Elias Efstathiou, Alessandra Spagnoli, Antonio Ciardi, Daniele Biacchi, John Spiliotis

**Affiliations:** 1 Department of Surgery “Pietro Valdoni”, Sapienza University of Rome, Rome, Italy; 2 Department of Surgery “Pietro Valdoni”, Azienda Policlinico Umberto, Rome, Italy; 3 First Department of Surgical Oncology, Metaxa Cancer Hospital, Pireaus, Greece; 4 Medical Oncology Unit, S. Andrew Hospital, Patras, Greece; 5 Department of Gynecology, Obstetric and Urological Science, Oncologic Day-Hospital, Sapienza University of Rome, Rome, Italy; 6 Department of Public Health and Infection Disease, Statistics Section, Sapienza University of Rome, Rome, Italy; 7 Department of Radiological Oncological and Pathological Sciences, Sapienza University of Rome, Rome, Italy

## Abstract

**Background:**

More information is needed for selection of patients with peritoneal metastases from endometrial cancer (EC) to undergo cytoreductive surgery (CRS) plus hyperthermic intraperitoneal chemotherapy (HIPEC).

**Methods:**

This study analyzed clinical, pathologic, and treatment data for patients with peritoneal metastases from EC who underwent CRS plus HIPEC at two tertiary centers. The outcome measures were morbidity, overall survival (OS), and progression-free survival (PFS) during a median 5 year follow-up period. Uni- and multivariate analyses were performed to identify significant factors related to outcome.

**Results:**

A total of 33 patients met the inclusion criteria and completed the follow-up period. At laparotomy, the median peritoneal cancer index (PCI) was 15 (range 3–35). The CRS procedure required a mean 8.3 surgical procedures per patient, and for 22 patients (66.6%), a complete cytoreduction was achieved. The mean hospital stay was 18 days, and major morbidity developed in 21% of the patients. The operative mortality was 3%. When surgery ended, HIPEC was administered with cisplatin 75 mg/m^2^ for 60 min at 43 °C. During a median follow-up period of 73 months, Kaplan–Meier analysis indicated a 5 year OS of 30% (median 33.1 months) and a PFS of 15.5% (median 18 months). Multivariate analysis identified the completeness of cytoreduction (CC) score as the only significant factor independently influencing OS. Logistic regression for the clinicopathologic variables associated with complete cytoreduction (CC0) for patients with metachronous peritoneal spread from EC who underwent secondary CRS plus HIPEC identified the PCI as the only outcome predictor.

**Conclusions:**

For selected patients with peritoneal metastases from EC, when CRS leaves no residual disease, CRS plus HIPEC achieves outcomes approaching those for other indications such as colon and ovarian carcinoma.

Endometrial cancer (EC) is the most common gynecologic cancer in developed countries.^1^ For low-risk patients (the majority), primary treatment achieves high overall cancer-specific survival rates,^2^,^3^ whereas for high-risk patients (20–30% of all those with a new diagnosis), treatment achieves low survival due to histologically aggressive tumors, adverse pathologic factors, and advanced disease at onset, inducing high recurrent disease rates and accounting for up to 50–60% of all EC-related deaths.^3^^–^6

Another common finding (10–30% overall incidence), especially among patients with recurrent high-risk EC, is intraperitoneal involvement, at a single disease site or combined with hematogenous or lymphatic spread.^4^,^7^,^8^ Although some reports could underestimate intraperitoneal spread through a transtubal route in early-stage disease,^9^,^10^ peritoneal disease generally involves patients with primary high-risk advanced-stage EC or those with recurrent tumors, both of whom have a dismal outlook.^3^,^11^,^12^

Ongoing trials currently are testing whether a combined approach using cytoreductive surgery (CRS) plus hyperthermic intraperitoneal chemotherapy (HIPEC), already standard care for selected patients with pseudomyxoma peritonei and peritoneal mesothelioma,^13^ improves outcome for peritoneal metastases from colorectal, gastric, and ovarian cancer, as well as for peritoneal surface malignancies (PSM) originating from other unusual sites.^14^,^15^ Although several retrospective series from centers experienced in treating PSM have analyzed the findings for synchronous or metachronous peritoneal metastases from EC treated with CRS and HIPEC, the small number of patients included preclude statistically reliable general conclusions.^16^^–^20 Further insights also are needed on prognostic factors and the criteria for selecting patients with peritoneal metastases from EC to undergo CRS and HIPEC.

We therefore investigated a series of patients who had peritoneal metastases from EC treated with CRS plus HIPEC at two tertiary centers experienced in treating PSM. We specifically aimed to assess the results of the integrated procedure achieved in this new PSM field to define outcomes and possible selection criteria more clearly.

The outcome measures were morbidity, overall survival (OS), and progression-free survival (PFS) during a median 5-year follow-up period. Uni- and multivariate analyses were performed to identify significant factors related to outcome.

## Methods

### Study Design

We conducted a retrospective multicenter cohort study of patients from two tertiary centers experienced in treating PSM. These patients underwent CRS plus HIPEC for peritoneal metastases from EC during the 14 years, from November 2002 to April 2016.

### Patient Population

Data were entered into a custom-designed database including only patients whose records contained complete information including age, Eastern Cooperative Oncology Group (ECOG) performance status, tumor markers, diagnostic techniques, International Federation of Gynecology and Obstetrics (FIGO) stage,^21^ tumor histology, peritoneal cancer index (PCI),^22^ surgical procedures and complications (Clavien-Dindo Classification),^23^ completeness of cytoreduction (CC) score,^22^ HIPEC techniques and drugs, in patients with metachronous peritoneal spread from EC, data regarding primary treatment, chemotherapy, eventual drug-induced toxicity during systemic chemotherapy and HIPEC evaluated with the National Cancer Institute Common Terminology Criteria for Adverse Events (CTCAE version 4.0),^24^ and last, complete, updated data on follow-up.

The indications for CRS and HIPEC were peritoneal metastatic spread from advanced or recurrent EC in patients younger than 75 years with adequate cardiac, renal, hepatic, and bone marrow function and ECOG performance status 0–2 with resectable disease who had signed written informed consent. The contraindications were extra-abdominal disease at CRS plus HIPEC, other malignancies, unresectable disease, and lack of fitness for the procedure. The two institutional review boards approved the study procedures before research activities started and prospective data collection began.

### Preoperative Management

Detailed staging depended chiefly on diagnostic imaging findings including computed tomography (CT), magnetic resonance imaging (MRI), and positron emission CT (PET-CT) combined with laparoscopy if imaging failed to specify resectability. Patients were scheduled for CRS plus HIPEC at a multidisciplinary meeting.

### CRS and HIPEC

At laparotomy, peritoneal spread was recorded according to the PCI.^22^ Patients then underwent CRS with peritonectomy procedures and visceral resections intended to leave no visible disease.^25^ For patients who had undergone laparoscopy, trocar sites were removed by full-thickness parietal resection. For patients with synchronous peritoneal spread, CRS included pelvic and paraaortic lymphadenectomy.^26^ Patients with metachronous peritoneal spread from EC underwent lymphadenectomy if it had not been performed previously, or if needed for evident nodal relapse. When surgery ended, HIPEC was administered with the closed technique.^27^

### Postoperative Management

All patients entered an intensive care unit (ICU) to receive prophylaxis for deep venous thrombosis and total parenteral nutrition until oral calorie intake became adequate. Morbidity was analyzed with the Clavien-Dindo classification,^23^ and operative mortality was defined as death within 90 days after surgery.

### Histopathology and Staging

Histopathology followed the new FIGO and World Health Organization (WHO) EC classifications including the dualistic classification proposed by Bokhman.^28^,^29^ All pathology slides underwent central review by a gynecologic pathologist (A.C.).

### Follow-up Evaluation

After CRS plus HIPEC, patients received adjuvant chemotherapy according to their general status and underwent follow-up evaluation according to a predefined scheme standardized for both institutions. For the first 2 years, asymptomatic patients were scheduled for clinical assessment and tumor marker testing every 3 months and diagnostic imaging every 6 months, and thereafter, clinical assessment and tumor marker testing every 6 months and yearly diagnostic imaging. In accordance with Esselen et al.^30^ recurrent disease sites after CRS plus HIPEC were classified as intraperitoneal, extraperitoneal, or distant. Extraperitoneal recurrences were defined as nodal, as were intraabdominal sites outside the peritoneal cavity, including intraparenchymal recurrences. Supradiaphragmatic non-nodal disease was classified as a distant recurrence.

### Statistical Analysis

Follow-up data were completed 31 December 2016. For continuous variables, we analyzed number of observations, median, and range, and for discrete variables, we analyzed number of observations and frequency. The Mann–Whitney *U* test was used to compare data in groups. In this study, OS was defined as the time from CRS plus HIPEC to the date of death from any cause, and PFS was defined as the time from CRS plus HIPEC to objective tumor progression or death from any cause, whichever occurred first. The study defined PFS2 as the time from CRS plus HIPEC to the second objective disease progression or death from any cause, whichever occurred first.^31^

Both OS and PFS probabilities were estimated using the Kaplan–Meier method. The log-rank test was used to compare between-group OS and PFS. The median OS and PFS and corresponding two-sided 95% confidence intervals (CIs) for each group were calculated using the Kaplan–Meier method. Univariate and Cox multivariate regression analysis model**s** were used to explore the influence of prognostic factors on OS and PFS. A logistic regression model was applied to evaluate whether clinicopathologic variables influenced CC scores. Statistical data were analyzed with the R statistical software package version 3.3.3. All *p* values lower than 0.05 were considered to indicate statistical significance.

## Results

The inclusion criteria were met by 36 patients attending the two participating tertiary centers who underwent CRS plus HIPEC. Of these 36 patients, 3 were lost to follow-up evalution. Of the remaining 33 patients, 5 had synchronous peritoneal metastases, whereas 28 had metachronous peritoneal metastases from EC and underwent primary or secondary CRS, both combined with HIPEC (Table 1). Three of the five patients for whom laparoscopy confirmed a PCI higher than 20 underwent six cycles of carboplatin AUC6 plus paclitaxel 175 mg/m^2^ every 3 weeks to lower tumor burden before primary CRS plus HIPEC and had a partial response (intraoperative PCI < 10). Of the remaining two patients, one refused NeoAdjuvant ChemoTherapy (NACT) and one had a low PCI (3) at diagnosis and directly underwent primary CRS plus HIPEC.

**Table 1:** Demographic and clinical characteristics of the 33 patients with peritoneal metastases from endometrial cancer (EC) undergoing cytoreductive surgery (CRS) plus hyperthermic intraperitoneal chemotherapy (HIPEC)

Variables	Peritoneal metastases from EC	
	CRS	
	Primary	Secondary
No. of patients	5	28
Age: years (range)	59 (42–65)	58 (43–73)
CA-125: U/ml (range)	150 (50–450)	230.5 (0–1500)
ECOG performance status		
0	4	12
1	1	15
Histology		
Type 1		
Endometrioid		
G2	1	3
G3	1	12
Type 2		
Serous	2	8
Clear cell	–	2
Carcinosarcoma	1	2
Squamous	–	1
Peritoneal cancer index (range)	9 (3–21)	16 (5–35)

Data are expressed as median and range unless otherwise stated

*ECOG* Eastern Cooperative Oncology Group

Of the 28 patients who underwent secondary CRS with a median of 17.5 months (range 6–36 months) elapsing after the first operation, 26 had previously undergone total hysterectomy and bilateral salpingo-oophorectomy with locoregional lymphadenectomy (21 pelvic and 5 combined pelvic and paraaortic lymphadenectomy). These 26 patients included 13 who had lymphatic spread at primary treatment and underwent adjuvant chemotherapy using two drugs (cisplatin and doxorubicin) in eight cases, three drugs (cisplatin, doxorubicin, and paclitaxel) in three cases. Two of these patients had a diagnosis of carcinosarcoma using ifosfamide combined with paclitaxel. Finally, four patients underwent vaginal brachytherapy, with two of the four patients receiving combined with adjuvant chemotherapy.

At laparotomy, the median PCI for the 33 patients was 15 (range 3–35), with no significant difference between the patients who underwent primary CRS and those who had secondary CRS (*p* = 0.09, Mann–Whitney *U* test). Overall, 273 surgical procedures were needed to achieve CRS for the 33 patients. All the patients who underwent primary CRS had pelvic and paraaortic lymphadenectomy, whereas after secondary CRS, 10 patients needed further lymphadenectomy for suspected nodal relapse. Surgery achieved complete cytoreduction (CC score, 0) for 22 patients (66.6%), whereas for 11 patients, it left residual disease as follows: CC1 for 7 patients (21.2%), CC2 for 3 patients (9.1%), and CC3 for 1 patient (3.1%). The mean hospital stay was 18 days, and major morbidity (grade 3 or 4) developed in 21% of the patients. The overall operative mortality was 3%, involving one patient who had an intraoperative massive pulmonary embolism (Table 2).

**Table 2:** Surgical procedures, outcomes, and morbidity

Surgical procedures	Cytoreductive surgery		
	Primary	Secondary	All patients
	(*n* = 5)	(*n* = 28)	(*n* = 33)
Peritonectomy			
Pelvic	4	18	22
Subtotal	1	10	11
Visceral resections			
Pelvic			
Histero-adnexectomy	5	–	5
Recurrent pelvic mass	–	20	20
Upper vaginectomy	–	7	7
Gastrointestinal			
Left colon	4	13	17
Right colon	–	4	4
Right+left colon	–	1	1
Transverse colon	–	–	–
Total colectomy	1	1	2
Small bowel	–	19	19
Appendectomy	4	14	18
Gastric	–	1	1
Hepatobiliary			
Cholecystectomy	2	7	9
Atypical hepatic resection	–	2	2
Pancreatic tail	–	1	1
Splenectomy	1	8	9
Genitourinary and others			
Partial bladder resection	–	3	3
Nephrectomy	–	1	1
Greater omentectomy	5	28	33
Round+falciform ligament	5	28	33
Implant resection/in situ destruction	1	25	26
Abdominal wall resection	5	9	14
Lymphadenectomy			
Regional	5	10	15
Total	43	230	273
Mean	8.6	8.2	8.3

Outcomes	Mean	Range
Duration of procedures (min)	375	120–660
Blood loss (ml)	600	100–900
Blood transfusions (U)	3	1–7
Plasma transfusions (U)	4	2–8
ICU stay (h)^a^	14	8–50
Postoperative stay (days)	18.6	9–90

Surgical morbidity grade^b^	*n*	%
1–2	10	33.3
3	5	15.1
4	1	3
5	1	3

*ICU* intensive care unit

^a^hours

^b^According to Clavien-Dindo classification

For 32 patients (excluding the patient who died intraoperatively of a massive pulmonary embolism), when surgical procedures ended, HIPEC was administered with a single drug (cisplatin 75 mg/m^2^) for 60 min at 43 °C. For two patients (6.2%), HIPEC induced a grade 1 or 2 acute kidney injury, which medical treatment promptly reversed.

At discharge, after a mean hospital stay of 48 days, 30 patients underwent multi-agent adjuvant chemotherapy variously integrated with biologic and molecular treatment for recurrent or metastatic disease. At this writing after a median follow-up period of 73-months (range 8–141 months; 95% CI 39.05–126.18 months), of the 32 patients who survived after CRS plus HIPEC, 8 are alive and disease free, 5 are alive with disease, and 19 have died.

**Fig. 1:**
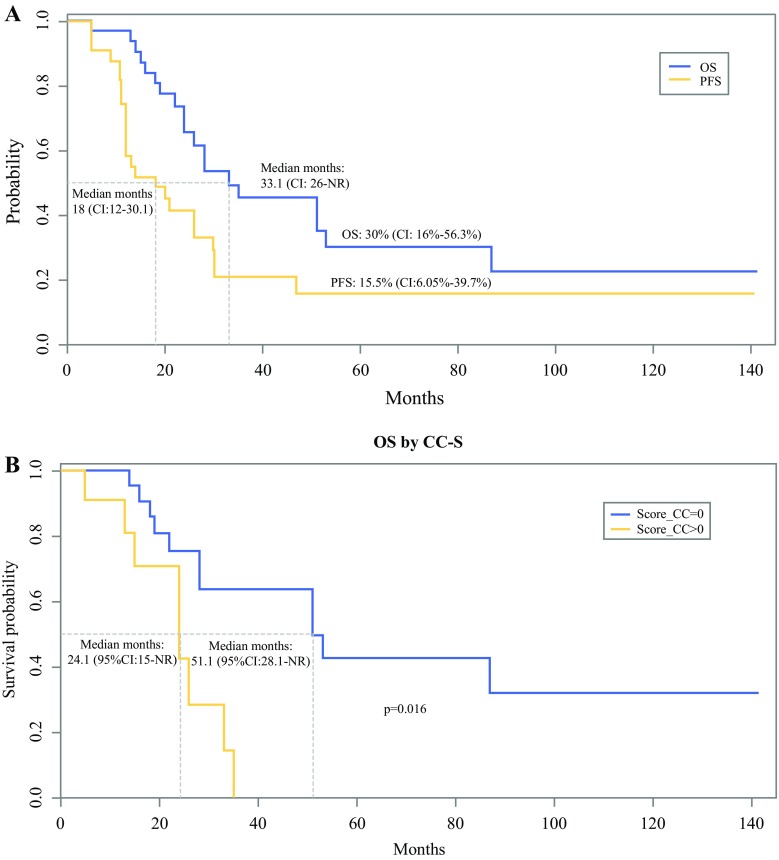
**a** Kaplan–Meier curves for overall survival (OS) and progression-free survival (PFS) of the 33 patients with peritoneal metastases from endometrial cancer who underwent cytoreductive surgery (CRS) plus hyperthermic intraperitoneal chemotherapy (HIPEC). **b** Kaplan–Meier curves for overall survival (OS) by the completeness of cytoreduction (CC) score of the 33 patients with peritoneal metastases from endometrial cancer who underwent CRS plus HIPEC

Of the 33 patients in this study, 24 had recurrent disease (involving the peritoneum in 54.1%) and received several chemotherapy regimens or further surgery (Table 3). Kaplan–Meier survival analysis showed a 5-year OS rate of 30% and a 5-year PFS rate of 15.5% (Fig. 1a). The median OS was 33.1 months (PFS 18 months; PFS2 28.2 months). The patients who underwent complete cytoreduction (CC0) had a significantly better OS than the patients whose surgery left residual disease (*p* < 0.016, log-rank test) (Fig. 1b).

**Table 3:** Clinical history of the 24 patients who had recurrent disease after cytoreductive surgery (CRS) and hyperthermic intraperitoneal chemotherapy (HIPEC)

Patient	1st Recurrence or progression			Treatment	2nd Recurrence or progression			Treatment	3rd Recurrence or progression			Treatment	Status
	Intra-peritoneal	Extra-peritoneal	Distant		Intra-peritoneal	Extra-peritoneal	Distant		Intra-peritoneal	Extra-peritoneal	Distant		
1	Peritoneal	–	–	CRS + HIPEC + Cht	–	–	Pleural Effusion	Pleurodesis + Cht	–	–	Lung	Cht	DOC
2	–	–	Pleural/lung	Pleurodesis + Cht	–	–	Lung	Palliation	–	–	–	–	DOD
3	Peritoneal	–	–	Palliation	–	–	–	–	–	–	–	–	DOD
4	–	–	Lung	Cht	–	–	Lung	Cht	–	–	–	–	AWD
5	–	–	Lung	Cht	–	–	Lung	Cht	–	–	Lung	Cht	AWD
6	–	Liver	Lung	Palliation	–	–	–	–	–	–	–	–	DOD
7	–	Nodal	Lung	Cht	–	Nodal	Lung	Cht	–	Nodal	Lung	Palliation	DOD
8	Peritoneal	Nodal	–	Palliation	–	–	–	–	–	–	–	–	AWD
9	–	–	Pleural/lung	Pleurodesis + Cht	–	–	Lung	Cht	–	–	–	–	AWD
10	Peritoneal	–	Pleural	Cht + pleurodesis	Peritoneal	–	–	Palliation	–	–	–	–	DOD
11	Peritoneal	Liver	–	Cht	Peritoneal	Liver	–	Palliation	–	–	–	–	DOD
12	–	–	Bone	Cht	–	–	Bone + pleural	Pleurodesis + Cht	–	–	Bone + lung	Palliation	DOD
13	Peritoneal	–	–	Palliation	–	–	–	–	–	–	–	–	DOD
14	Peritoneal	–	–	CRS + HIPEC	Peritoneal	–	–	Palliation	–	–	–	–	DOD
15	–	Liver	–	Cht	–	Liver	–	Cht	–	Liver	–	Palliation	DOD
16	Peritoneal	–	–	Cht	Peritoneal	–	–	Palliation	–	–	–	–	DOD
17	–	–	Pleural	Pleurodesis + Cht	–	–	–	–	–	–	–	–	DOC
18	–	Nodal	–	Cht	–	Nodal	Palliation	–	–	–	–	–	DOD
19	–	Liver	–	Cht	–	Liver	Palliation	–	–	–	–	–	DOD
20	Peritoneal	Nodal	–	Cht	Peritoneal	–	–	Palliation	–	–	–	–	DOD
21	Peritoneal	–	–	Cht	Peritoneal	–	–	Cht	–	–	–	–	AWD
22	Peritoneal	–	–	Cht	Peritoneal	–	–	Palliation	–	–	–	–	DOD
23	Peritoneal	Nodal	–	Cht	Peritoneal	Nodal	–	Palliation	–	–	–	–	DOD
24	Peritoneal	–	–	Cht	Peritoneal	–	–	Palliation	–	–	–	–	DOD

*Cht* systemic chemotherapy, *DOC* died of other causes, *DOD* died of disease, *AWD* alive with disease

The univariate analysis (log-rank test) for OS identified the CC score and PCI as the only two factors significantly influencing outcome. The univariate analysis for PFS identified the PCI as the only significant factor. The multivariate Cox regression analysis reevaluating significant univariate prognostic factors identified the CC score as the only significant factor capable of independently influencing OS (Table 4). Logistic regression for the clinicopathologic variables associated with complete cytoreduction (CC0) in the 28 patients with metachronous peritoneal spread from EC who underwent secondary CRS combined with HIPEC identified the PCI as the only outcome predictor (OR 1.24; 95% CI 1.09–1.53). A one-unit increase in PCI value increased the risk of a CC score higher than 0 by 24%.

**Table 4:** Prognostic factors for progression-free survival (PFS) and overall survival (OS) of the patients with peritoneal metastases from evaluable endometrial cancer (*n* = 32) (Cox proportional-hazards model)

Variables	Survival analysis		
	Univariate		Multivariate
	PFS	OS	OS
	HR (95% CI)	HR (95% CI)	HR (95% CI)
Age at diagnosis	1.01 (0.97–1.06)	1.02 (0.97–1.08)	–
CC score			
0	–	–	–
> 0	1.90 (0.82–4.41)	3.94 (1.39–11.18)	3.92 (1.39–11.12)
Histology type			
1	–	–	–
2	1.52 (0.64–3.43)	1.16 (0.47–2.86)	
PCI	1.06 (1.01–1.12)	1.06 (1.01–1.13)	–

*HR* hazard ratio, *CI* confidence interval, *PFS* progression-free survival, *OS* overall survival, *CC* completeness of cytoreduction, *PCI* peritoneal cancer index (considered as a continuous variable)

## Discussion

This series of patients who had peritoneal metastases from EC treated with CRS plus HIPEC at two tertiary centers experienced in treating PSM was relatively large given that peritoneal metastases from EC involve an unusual site for this combined treatment. Despite the rare indications and given that treating these patients is a challenging task, overall, we obtained with acceptable morbidity, outcome rates generally approaching those reported for CRS plus HIPEC in other indications.^32^^–^34

Our findings in this series of patients with peritoneal metastases from EC treated with CRS plus HIPEC are hard to compare with others. Although two collective reviews report a series of patients who underwent combined treatment for peritoneal metastases from various primary diseases including EC, they failed to analyze the features of these patients in detail.^15^,^33^ The only homogeneous comparison is in the series of 13 patients treated by Delotte et al.^19^ These investigators reported a complete cytoreduction rate analogous to that in our series, but achieved higher overall survival rates also if the median PCI and median follow-up period were less than the rates we report (median PCI, 12 vs. 15; median follow-up period, 19.4 vs. 73 months).

An equally difficult task was to compare our patients who had advanced or recurrent EC with peritoneal spread treated using CRS plus HIPEC with other patients who underwent CRS alone. In the past few years, therapeutic advances have recommended (e.g., for ovarian cancer)^35^ maximal cytoreduction aimed at leaving no residual disease as the cornerstone of every multimodal therapeutic strategy.^36^^–^44 Adjuvant chemotherapy unfortunately seems poorly effective for patients with advanced high-risk EC,^3^,^6^ leading to considerably lower outcome rates than those obtained for ovarian cancer.^45^ Precise identification of metastatic peritoneal disease and quantification of its extent are factors rarely considered by major case series analyzing the results obtained with CRS alone for primary advanced or recurrent EC. Moreover, when studies refer to peritoneal spread, they usually do so to underscore that this is a typical site for metastatic spread in high-risk patients, worsened by an unfavorable outcome.^4^,^6^,^8^ Hence, comparison of outcome findings for our patients treated using CRS plus HIPEC with those for patients who undergo CRS alone suffers from bias. Bias apart, our outcome results compare well with published data, especially for those patients in whom CRS combined with HIPEC achieved complete cytoreduction.^12^,^37^,^38^,^41^,^42^,^45^,^46^

Despite a few exceptions,^6^,^46^ in our series, compared with most published series analyzing data for CRS alone in treating advanced or recurrent EC,^37^,^38^,^41^,^42^ the only independent prognostic factor able to predict outcome was the completeness of cytoreduction. Although it remains conjectural whether HIPEC improved outcome in our series, an outcome finding that emerged from analysis of the 24 patients who had recurrent disease was the site of recurrence. Only 50% of the recurrent disease sites involved the peritoneum, thus implying that HIPEC might act as a protective factor. This finding supports what Esselen et al.^30^ have already reported for advanced ovarian cancer treated after CRS with endoperitoneal normothermic adjuvant chemotherapy.

Our series also provided some help in identifying the criteria for selecting CRS plus HIPEC to manage peritoneal metastases from EC. For this purpose, we need to distinguish synchronous from metachronous peritoneal metastases. Despite the few synchronous peritoneal metastases, NACT reduced peritoneal involvement before CRS for three of our five patients, and achieved long-term PFS for the remaining two patients. Given that others have used NACT in primary advanced EC for no more than 100 patients, it still seems premature to consider NACT responses as a selection criterion for CRS with HIPEC.^47^ For the 28 patients with metachronous peritoneal metastases from EC, the logistic regression analysis testing clinicopathologic factors associated with complete cytoreduction identified PCI as the only outcome predictor. A possible PCI cutoff value for selecting patients to undergo CRS with HIPEC remains open for future research in a larger cohort.

Because none of our patients with advanced-stage EC had undergone genomic characterization to identify molecular biomarkers predicting individual tumor behavior, we cannot say to which molecular EC subgroup their tumors belonged.^48^ Nor can we say whether molecular status explained the wide survival range. Future research should improve the emerging molecular classification tools for risk group assessment and avoid wasting resources by identifying cost-effective molecular-targeted therapy.^49^

The limitations of our study were its retrospective design and the small number of patients enrolled. During the natural history of EC, only 15–30% of patients have peritoneal metastatic spread, precluding prospective randomized-controlled trials.^39^

In conclusion, for patients with peritoneal metastases from EC, especially when CRS leaves no residual disease, CRS plus HIPEC administered in experienced centers for properly selected cases achieves OS and PFS outcome rates approaching those for the most frequent indications for this combined procedure (colorectal or ovarian cancer).^32^,^34^,^50^ Given that positive peritoneal cytology is a risk factor for peritoneal relapse and that some suggest including omentectomy in staging high-risk EC,^9^,^11^,^26^,^51^ HIPEC plus CRS might help to prevent peritoneal spread in EC.
